# Negative Aspects of the Regional Quota System in Japan

**DOI:** 10.31662/jmaj.2018-0055

**Published:** 2019-02-22

**Authors:** Kana Yamamoto, Akihiko Ozaki, Morihito Takita, Tomohiro Morita, Hiroaki Saito, Yuki Senoo, Tetsuya Tanimoto, Masahiro Kami

**Affiliations:** 1 Medical Governance Research Institute, Tokyo, Japan

**Keywords:** physician maldistribution, medical school, student loan, postgraduate medical education

## Abstract

*Chiikiwaku* is a measure to improve the maldistribution and shortage of physicians in rural areas in Japan. Although *Chiikiwaku* quota seats have been on the rise, a considerable number of young physicians and medical students return their loans and do not fulfill their duty periods for several important reasons, including an obligation term lasting for 9 years. The number of medical students who do not apply for admission to the *Chiikiwaku* quota has increased, and vacancies have developed in some medical schools. Urgent modification of this program is therefore required to make it suitable for actual situations of both rural medical care and the education of young physicians.

The unequal geographical distribution of doctors is a serious problem in several developed countries. Doctors concentrate in urban cities, and their shortage is serious in rural areas ^(1)^. The Japanese government has taken various measures to improve this situation.

One of these measures is *Chiikiwaku*, a program combining a student loan and a regional admission quota ^(1)^. All applicants have to be engaged in the medical practice in rural areas falling under their alma mater’s prefecture immediately upon graduation.

Since the introduction of this program in 2009, *Chiikiwaku* quota seats have been on the rise. According to the Ministry of Education, Culture, Sports, Science and Technology, these seats reached 1,674 in 2017, accounting for 20% of all the medical school enrollees (8,279). Some researchers reported that this program has contributed to alleviating the physician maldistribution in Japan ^(1)^. Yet, the limitations of this program have not been sufficiently acknowledged.

The most serious problem is that the obligation term is as long as 9 years. To leave the program, enrollees have to repay the entire loan amount taken for meeting the cost of the program. The amount, which covers the enrollment fee, tuition fees, and living expenses, reaches approximately USD 100,000 over a 6-year student period ^(2)^. The interest rate associated with this loan is higher than 10% in some prefectures, and students also have to repay USD 80,000 as the interest cost ^(2)^.

Nonetheless, a number of young doctors do not fulfill their duty periods, preferring to return their loans. According to the Ministry of Health, Labor and Welfare (MHLW), 123 of 5,290 medical students (2.3%) enrolled in this program withdrew from it during 2008–2018. MHLW reported that the number of students leaving *Chiikiwaku *would increase to 7% in the future. In 2018, the proportion of vacancy seats exceeded 20% of the total seats in the *Chiikiwaku* programs of 22 medical schools. The *Chiikiwaku* program has not functioned in an actual clinical setting.

The Japanese government raised concerns about the withdrawal of several young doctors from the *Chiikiwaku* program and strengthened regulation by delivering a notice to hospital directors not to hire physicians who left this program. As a result, only 9 of 805 doctors (1.1%) who were enrolled in the* Chiikiwaku* program withdrew from it in 2018. However, such a compulsive approach of MHLW has been pointed out as being illegal ^(2)^, and there is a problem concerning its sustainability. If doctors enrolled in the *Chiikiwaku* program are forced to engage in medical practice in rural areas, they might quit the job ^(3)^.

It is imperative to overcome the limitations of this system and make it sustainable. As a model case that can be emulated, we would like to present a US program. Students admitted to the University of Kansas School of Medicine are eligible to apply for the Kansas Medical Student Loan program, which gives them a maximum of USD 2,000 per month ^(4)^. The loan is offered to those who are determined to pursue a career in primary care. Despite its similarity with the *Chiikiwaku* program in making applicants who fail to meet the obligations of this program repay their loans at 15% interest, it is more flexible than the Japanese program in terms of career choice ^(4)^. For instance, applicants are allowed to finish the residency program before they engage in their practice, which is the return on the program. The scheme also enables program enrollees to attain a certification. Furthermore, the period of compulsory practice under the program obligation is approximately 9–12 months, which is shorter than that in Japan’s regional quota system program ^(2)^. In *Chiikiwaku*, the period of compulsory practice under the program is 3–12 years, and a 9-year duration is the most popular.

We realize that it may be difficult to directly import this example into Japan, given the differences in the medical education system in the two countries. However, the importance of postgraduate medical education should at least be taken into account when designing a regional quota system. There is a case where the number of physicians has increased in a hospital in a remote area in Japan without relying on *Chiikiwaku* ^(5)^. In this respect, a good starting point for reform of the program could be to offer it only to those medical students who are determined to engage in primary care in the future.

## Article Information

### Conflicts of Interest

Dr. Yamamoto received personal fees from NAGATANIEN Co., Ltd. and ROHTO Pharmaceutical Co., Ltd., outside of the submitted work; Dr. Ozaki received personal fees from MNES Inc., outside of the submitted work; Dr. Tanimoto received personal fees from MNES Inc., outside of the submitted work; Dr. Saito received personal fees from TAIHO Pharmaceutical Co., Ltd., outside of the work; Dr. Masahiro Kami received a donation from AIN HOLDINGS Inc. and is remunerated as outside director of SBI Biotech Co., Ltd., SBI Pharmaceuticals Co., Ltd., and Y’s Inc.; he is also an advisor of MNES Inc. and MRSO Inc.

### Author Contributions

All authors conceived and designed the study and are responsible for the integrity and accuracy of the data. KY, AO, MT and MK drafted the manuscript. All authors critically reviewed the manuscript for important intellectual content and provided final approval.
